# Harnessing uncertainty in radiotherapy auto-segmentation quality assurance

**DOI:** 10.1016/j.phro.2023.100526

**Published:** 2023-12-19

**Authors:** Kareem A. Wahid, Jaakko Sahlsten, Joel Jaskari, Michael J. Dohopolski, Kimmo Kaski, Renjie He, Enrico Glerean, Benjamin H. Kann, Antti Mäkitie, Clifton D. Fuller, Mohamed A. Naser, David Fuentes

**Affiliations:** a Department of Imaging Physics, The University of Texas MD Anderson Cancer Center, Houston, TX, USA; b Department of Radiation Oncology, The University of Texas MD Anderson Cancer Center, Houston, TX, USA; c Department of Computer Science, Aalto University School of Science, Espoo, Finland; d Department of Radiation Oncology, The University of Texas Southwestern Medical Center, Dallas, TX, USA; e Department of Neuroscience and Biomedical Engineering, Aalto University School of Science, Espoo, Finland; f Artificial Intelligence in Medicine Program, Brigham and Women’s Hospital, Dana-Farber Cancer Institute, Harvard Medical School, Boston, MA, USA; g Department of Otorhinolaryngology, Head and Neck Surgery, University of Helsinki and Helsinki University Hospital, Research Program in Systems Oncology, University of Helsinki, Helsinki, Finland

We have read with great interest the article by Outeiral et al. [1], in which the authors propose a simple metric for optimizing quality assurance in deep learning (DL) auto-segmentation workflows. We commend the authors for their insightful analysis using two meticulously curated MRI datasets. Through this study, the authors have probed into the relatively underexplored yet clinically relevant domain of uncertainty estimation in auto-segmentation.

One of the key contributions of this study is the reappropriation of standard DL outputs as a quality indicator to identify cases that clinicians should review further. The authors achieve this by applying an empirically derived threshold to the softmax output of their DL network, computing the mean of the thresholded score map (termed the HiS metric), and correlating it with standard geometric quality indices. When juxtaposed with a mean entropy — a commonly used measure of model output uncertainty — HiS consistently demonstrated a stronger correlation with the geometric indices, suggesting its superior ability to stratify cases needing additional review. We applaud the authors' efforts for their novel contributions and would like to note some potential caveats that could pave the way for future research directions.

Conventional large DL networks often yield overconfident predictions which can result in poor model calibration [2], meaning the predicted probabilities do not align with the true underlying data. This discrepancy could undermine the reliability of these outputs in detecting out-of-distribution data, a critical aspect of quality assurance systems. Notably, the direct use of softmax outputs as measures of model uncertainty is a point of contention within the DL community [3], [4]. However, moderately sized standard DL networks have the potential to exhibit well-calibrated performance [5]. Therefore, it is unclear whether calibration had a major impact on Outeiral et al.’s analysis using a standard ​​nnU-Net architecture. In contrast, Bayesian DL approaches have been observed to be well-calibrated and may circumvent these issues [6]. Specifically, the application of approximate Bayesian techniques, such as Monte Carlo dropout [7] or deep ensembles [8] (Fig. 1), is relatively simple compared to conventional solutions. While these methods demand a slightly higher computational cost, they could be considered for investigating HiS in future studies. Importantly, ensembling (e.g., through cross-validation schemes) is becoming increasingly common for many DL solutions [9]. We have previously benchmarked ensembling under a U-net framework for uncertainty estimation in oropharyngeal cancer auto-segmentation and have shown its efficacy [10]. Interestingly, Outeiral et al. use cross-validation within their study for robustness analysis; merging their cross-validation outputs into an ensemble could have improved calibration when employing their HiS metric. Of note, alternative methods that allow for calibrated uncertainty estimates, such as conformal prediction [11], may also show promise for auto-segmentation and should be further investigated.

**Fig. 1:**
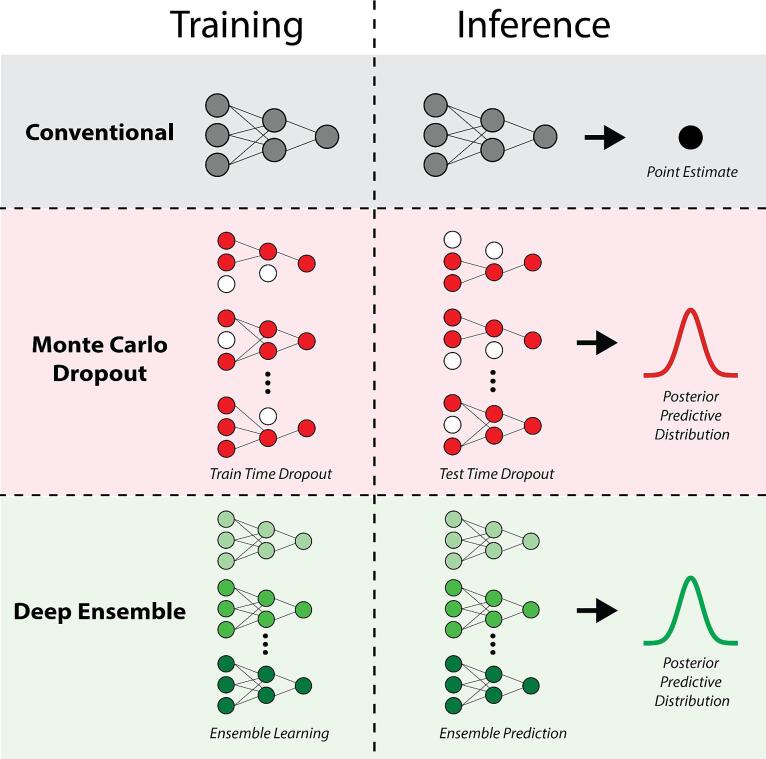
Comparison between conventional and approximate Bayesian deep learning approaches. Conventional deep learning methods generate point estimates and are often poorly calibrated, while approximate Bayesian methods, e.g., Monte Carlo dropout and deep ensemble, generate posterior predictive distributions that are often better calibrated. The Monte Carlo dropout method consists of randomly removing nodes from the network during the training and inference procedures. The deep ensemble method trains submodels with different random initializations of network parameters and, optionally, varying subsets of training data, then combines their predictions. This figure is loosely inspired by figures from van den Berg and Meliadò [14].

Finally, we would like to note that the proposed HiS metric, if used to measure uncertainty, may be unable to disentangle epistemic uncertainty (i.e., intrinsic model uncertainty) and aleatoric uncertainty (i.e., extrinsic statistical uncertainty) [12]. While the same can be said of general measures of entropy, there exist alternative entropy-related uncertainty metrics, like expected entropy and mutual information, that could distinguish the source of the uncertainty when combined with an approximate Bayesian approach [10], [13]. Moreover, when the distribution of DL network parameters is assumed to be a delta distribution, e.g., in a conventional DL network, the epistemic uncertainty is implicitly assumed to be non-existent. Therefore, depending on the specific auto-segmentation use case, alternative uncertainty metrics, or combinations of uncertainty metrics, may be more suitable.

An increasing number of studies have begun to apply uncertainty estimation to the quality assurance of radiotherapy-related auto-segmentation [10], [14], [15], [16], [17], [18], [19], [20], [21], [22], [23]. The study by Outeiral et al. serves as a cornerstone contribution to this crucial literature. We eagerly anticipate further advances in this clinically significant field of work.

## 1 Declaration of generative AI and AI-assisted technologies in the writing process

During the preparation of this work, the authors used ChatGPT (GPT-4 architecture; ChatGPT October 17, 2023 Version) to improve the grammatical accuracy and semantic structure of portions of the text. After using this tool, the authors reviewed and edited the content as needed and take full responsibility for the content of the publication.

## 2 Funding Acknowledgements

Kareem A. Wahid is supported by the NCI NRSA Image Guided Cancer Therapy Training Program (T32CA261856). The work of Joel Jaskari, Jaakko Sahlsten, and Kimmo K. Kaski was supported in part by the Academy of Finland under Project 345449. Antti Mäkitie is supported in part by a grant from the Finnish Society of Sciences and Letters. Benjamin H. Kann is supported by an NIH/National Institute for Dental and Craniofacial Research (NIDCR) K08 Grant (K08DE030216). Clifton D. Fuller receives related grant support from the NCI NRSA Image Guided Cancer Therapy Training Program (T32CA261856), as well as additional unrelated salary/effort support from NIH institutes. Clifton D. Fuller also receives grant and infrastructure support from MD Anderson Cancer Center via: the Charles and Daneen Stiefel Center for Head and Neck Cancer Oropharyngeal Cancer Research Program; the Program in Image-guided Cancer Therapy; and the NIH/NCI Cancer Center Support Grant (CCSG) Radiation Oncology and Cancer Imaging Program (P30CA016672). David Fuentes was supported by R01CA195524.

## Declaration of competing interest

Clifton D. Fuller has received unrelated direct industry grant/in-kind support, honoraria, and travel funding from Elekta AB.
